# Evaluation of an Australian health literacy training program for socially disadvantaged adults attending basic education classes: study protocol for a cluster randomised controlled trial

**DOI:** 10.1186/s12889-016-3034-9

**Published:** 2016-05-27

**Authors:** Kirsten J. McCaffery, Suzanne Morony, Danielle M. Muscat, Sian K. Smith, Heather L. Shepherd, Haryana M. Dhillon, Andrew Hayen, Karen Luxford, Wedyan Meshreky, John Comings, Don Nutbeam

**Affiliations:** Sydney School of Public Health, The University of Sydney, Sydney, NSW 2006 Australia; Centre for Medical Psychology and Evidence-based Decision-making (CeMPED), Sydney School of Public Health, The University of Sydney, Sydney, NSW 2006 Australia; Psychosocial Research Group, Prince of Wales Clinical School, Faculty of Medicine, University of New South Wales, Sydney, NSW 2052 Australia; Psycho-oncology Co-operative Research Group (PoCoG), School of Psychology, The University of Sydney, Sydney, NSW Australia; Centre for Medical Psychology and Evidence-based Decision-making (CeMPED), School of Psychology, The University of Sydney, Sydney, NSW 2006 Australia; Concord Clinical School, The University of Sydney, Sydney, NSW 2006 Australia; School of Public Health and Community Medicine, University of New South Wales, Sydney, NSW 2052 Australia; Patient Based Care, Clinical Excellence Commission, Sydney, NSW 2000 Australia; NPS Medicinewise, Surry Hills, NSW 2010 Australia; Center for International Education, University of Massachusetts, Amherst, MA 01003 USA

**Keywords:** Literacy, Health literacy, Adult education, Shared decision making, Numeracy, Health disparities, Minority health, Underserved patients, Social inequality, Social disadvantage

## Abstract

**Background:**

People with low literacy and low health literacy have poorer health outcomes. Literacy and health literacy are distinct but overlapping constructs that impact wellbeing. Interventions that target both could improve health outcomes.

**Methods/design:**

This is a cluster randomised controlled trial with a qualitative component. Participants are 300 adults enrolled in basic language, literacy and numeracy programs at adult education colleges across New South Wales, Australia. Each adult education institute (regional administrative centre) contributes (at least) two classes matched for student demographics, which may be at the same or different campuses. Classes (clusters) are randomly allocated to receive either the health literacy intervention (an 18-week program with health knowledge and skills embedded in language, literacy, and numeracy training (LLN)), or the standard Language Literacy and Numeracy (LLN) program (usual LLN classes, specifically excluding health content).

The primary outcome is functional health literacy skills – knowing how to use a thermometer, and read and interpret food and medicine labels. The secondary outcomes are self-reported confidence, more advanced health literacy skills; shared decision making skills, patient activation, health knowledge and self-reported health behaviour. Data is collected at baseline, and immediately and 6 months post intervention. A sample of participating teachers, students, and community health workers will be interviewed in-depth about their experiences with the program to better understand implementation issues and to strengthen the potential for scaling up the program.

**Discussion:**

Outcomes will provide evidence regarding real-world implementation of a health literacy training program with health worker involvement in an Australian adult education setting. The evaluation trial will provide insight into translating and scaling up health literacy education for vulnerable populations with low literacy.

**Trial registration:**

Australian New Zealand Clinical Trials Registry ACTRN12616000213448.

**Electronic supplementary material:**

The online version of this article (doi:10.1186/s12889-016-3034-9) contains supplementary material, which is available to authorized users.

## Background

### Low literacy and health literacy

Health literacy is commonly defined as the *capacity to acquire, understand and use information in ways which promote and maintain good health* [1, 2]. As with general literacy, health literacy skills can be described and measured at different levels: “functional” (the health skills required to function in everyday situations), “communicative/interactive” (more advanced skills to extract information, discriminate between different sources of information and derive meaning) and “critical” (the ability to critically analyse information) [3, 4]. Health literacy involves the capacity to *use* health information, not simply to obtain it [3].

To the extent that health literacy involves engaging with written information, it is strongly influenced by language, literacy and numeracy (LLN); hence some commonly used measures of health literacy (e.g. REALM [5], TOFHLA [6]) measure recognition and use of health vocabulary. Individuals with limited literacy skills are less likely to engage in preventative healthcare, are more likely to develop chronic illnesses [7] and, once developed, experience greater difficulties managing those illnesses and often die earlier [8]. In Australia, up to 60 % of adults lack basic health literacy skills to understand health-related materials, such as instructions on a medicine label [9]. Estimates of low health literacy in Europe (47 %) [10] and the US (36 %) [11] are similarly high. Building health literacy is a priority in Australia and internationally [10, 12–14].

Closely aligned with emerging interest in population heath literacy has been a growing recognition of the value of Shared Decision Making in health care. Shared Decision Making occurs when patients and health professionals work together to jointly make decisions about a patient’s health [15, 16]. It is a cornerstone of high quality healthcare and now endorsed by major health organisations internationally [13, 17, 18]. Research shows that patients who share health decisions have higher health knowledge, more accurate risk perceptions, reduced difficulty with decision-making [19, 20], and in some circumstances show improved clinical outcomes [21–23]. Shared Decision Making and involvement in health care generally may be viewed within Nutbeam’s conceptual framework as “communicative” and “critical” health literacy. Yet there has been relatively little effort to support Shared Decision Making for adults with low general literacy and it has not been incorporated into health literacy programs to date [24]. There is a need to both develop and test health literacy interventions that support Shared Decision Making for adults with lower levels of general literacy.

### Health literacy interventions

Partnerships between health and education agencies offer promise in building health literacy in the population [25], especially if they collaborate to access hard-to-reach groups and develop strategies to improve both health literacy, and general language, literacy, and numeracy (LLN) skills [26, 27]. The field of adult literacy has been cautious about integrating health content into adult education programs due to concerns that teachers may lack the expertise and confidence to deliver topics about health [28]. This is changing, although rigorous evaluations are lacking.

In the US, the Health Literacy Study Circles is a program providing teachers tools to develop health-related units, lessons and action plans [29]. The program as part of a broader intervention has been reported to increase students’ health-related knowledge and self-efficacy [30]. Three ‘health literacy’ style programs have been run in adult education settings and evaluated in randomised trials in the US [31–33]. Although published work has indicated some degree of success of these programs [32], only one was published in a peer reviewed journal [33] making it difficult to ascertain how effective these programs have been. The one published study, a trial of a community-based health literacy/English as a Second Language program was targeted at Hispanics in the United States. It showed promising increases in health-related verbal fluency (using the TOFHLA) in the health literacy arm [33] immediately post intervention but there was no further follow up and measures focused on a functional health literacy only.

A UK based program that embedded health content into adult basic education was the *Skilled for Health* program [34]. It found that health content helped to engage and retain socially disadvantaged adult learners and participants reported improved understanding about health and healthier behaviours (diet, exercise and smoking). However, improvements in specific health literacy skills were not directly assessed and health literacy and health behaviour outcomes were measured by self-report. Additionally the study did not use a randomised design and lacked a control group for rigorous comparison [34].

### The Australian health literacy program

We aim to evaluate the efficacy of an intervention similar to Skilled for Health, adapted for an Australian context using a randomised trial design, with outcomes assessed immediately post intervention and at 6 months to determine whether any health literacy gains are retained.

A novel addition to the program in this study is the development and inclusion of a Shared Decision Making component (see [35]). This seeks to teach students about the concept of Shared Decision Making (i.e. teaching them that there are often different test and treatment options, that they have a choice between options, and that they have the right to ask questions); provide education about probability and risk, (i.e. how to understand the outcomes and likelihood of different options); and develop self-efficacy to express preferences and be actively involved in health decisions. Specifically, we introduced the AskShareKnow questions [36] to students (What are my options? What are the possible benefits and harms of those options? How likely are each of those benefits and harms to happen to me?) as a generic Shared Decision Making tool to help achieve these aims. The AskShareKnow questions have been shown to increase the amount and quality of information about treatment options provided by family physicians and increase Shared Decision Making behaviours in consultations [36], and the demonstration of these questions via a short video clip in a clinic waiting room was found to be a feasible approach to increase question asking in health care consultations [37].

The population of adults attending basic LLN classes in Australia comprises two distinct groups: native English speakers (mostly Australian-born) who may have experienced a range of learning disabilities, did not complete school, or are retraining as a condition of their unemployment benefits; and (migrants) from culturally and linguistically diverse backgrounds (CALD) who may fit any or none of the above conditions. Some of these migrants are highly-educated, but have poor (oral and/or written) English skills; others may have never attended school. The challenge is to develop a program flexible enough to cater to the different needs of these populations and deliver measureable differences in health literacy outcomes.

### Study objectives

This is a cluster randomised controlled trial that aims to evaluate a best practice model to:

- Deliver improvements in health literacy, confidence in health skills, Shared Decision Making, and knowledge about health and healthy behaviour, through a health literacy education program.
- Demonstrate improved engagement in learning (through higher attendance at classes) and greater enrolment in further adult education classes.
- Assess feasibility for delivery in a variety of locations (metropolitan, regional, and remote).

The health literacy program will be compared to a standard adult language, literacy and numeracy (LLN) program which would be expected to achieve some improvements which extend to health literacy outcomes also. Therefore this trial will examine the added benefit the health literacy program brings to health literacy related outcomes.

## Methods

### Study design

**Fig. 1:**
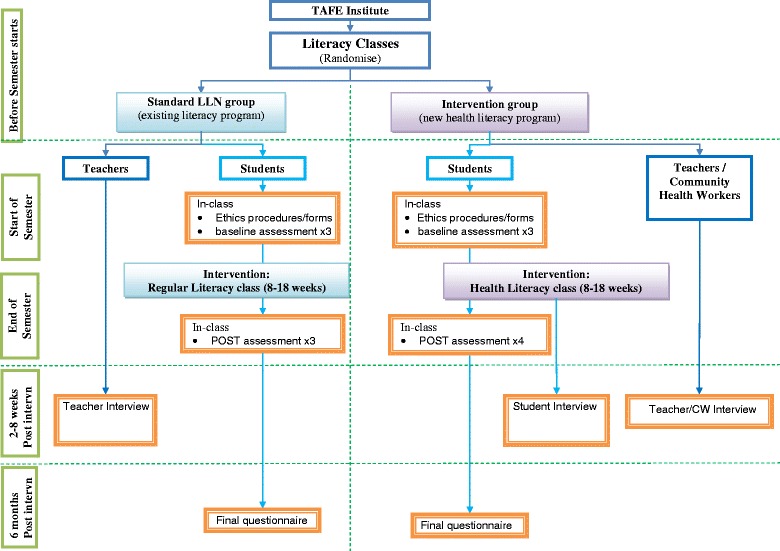
Flowchart of study activities

This study is a matched cluster randomised controlled trial (Fig. 1), in which a class represents a cluster. Each participating institute will be required to provide (at least) one matched pair of classes, in which a pair comprises one intervention and one standard LLN class. The two classes within each pair will be a priori matched for typical student demographics (e.g. day or evening students; metropolitan or regional; predominance of native English or NESB students) and enrolled in the same course code.

Intervention and standard LLN classes will have the same TAFE-specified units of competency to cover. Students in the health literacy (“intervention”) classes will learn literacy and numeracy skills with content focused on health topics; the standard LLN classes will cover the same literacy and numeracy skills, in contexts other than health. These “standard LLN” classes control for both expected literacy gains as well as any research participation effects [38].

### Ethics and dissemination

The study has ethical approval from the University of Sydney Human Research Ethics Committee and 10 NSW Institutes of TAFE. Results will be published in peer reviewed journals, and reported to partner organisations. Written informed consent will be obtained from all participants.

### Recruitment

Students and teachers will be recruited from TAFE institutes in NSW, Australia. There are 10 Institutes of TAFE in NSW – each one will be invited to join the study for a full (18-week) semester, and to contribute at least one matched pair of classes delivering the same TAFE course (in the same or different location).

All students enrolled in each participating class will be invited to participate in the study during the first class. To assist recruitment, the researchers (KM, SM, DM) created a video introducing themselves and explaining the purpose of the study.

Participating classes will be basic LLN courses corresponding to Level 2 (basic/beginner) of the 5-level Australian Core Skills Framework (ACSF). The ACSF describes performance on the 5 skills of learning, reading, writing, oral communication, and numeracy, and is assessed by TAFE teachers at student enrolment. Each TAFE course has a designated code, and specifies LLN learning outcomes that students must achieve. For relevant course codes, a “mapping” document produced by a TAFE coordinator indicates which units of the health literacy program cover each LLN competency.

### Randomisation

At enrolment, students will be allocated to classes following normal TAFE procedures. Classes will be randomised centrally into “intervention” or “standard LLN” groups by the University of Sydney team. At campuses holding only one class participating in the study, all enrolling students will be allocated to that class. At larger metropolitan campuses with more than one participating class, students will be randomised into classes on enrolment day, by selecting an “odd” or “even” paper ticket. Matched classes at the same location will be held on different days to minimise risk of contamination between intervention and standard groups.

### Participants and setting

Participants will be recruited following standard TAFE processes for enrolling students. Participating TAFE teachers will invite 300 adults assessed at ACSF level 2, seeking basic education at TAFE in NSW to join the study. As part of standard procedures, students’ literacy and numeracy levels are assessed by TAFE teachers on enrolment day.

### Participating sites

We will recruit classes from TAFE Institutes in metropolitan and regional centres across NSW, Australia, including multiple Sydney districts, and Illawarra, Hunter, New England, and Southern NSW.

### Inclusion criteria

Eligible classes will be learning basic language, literacy and numeracy at ACSF level 2 (in one of 4 eligible course codes). Intervention and standard LLN classes will be matched on typical demographic characteristics including age, sex, English proficiency, literacy skills, course code, and region. Students will be 16 years and older. Consent from a parent or guardian must be obtained for students under 18 years.

### Intervention: the health literacy program

Australian adult education and public health experts adapted the UK *Skilled for Health* program to an Australian context. The revised program embeds key LLN skills development at the Australian Core Skills Framework level 2 into materials containing health-related topics focused on public health priorities (as identified by NSW Health) using Functional Context Education (FCE) methods [39]. In this approach students learn specified LLN skills through engaging with health material guided by their teacher [40]. We added a Shared Decision-Making program centred around the AskShareKnow questions (as described in [35]) that teaches participants about participating in decision making about their health, using recommended questions to elicit information from healthcare professionals, and discussing how to understand their responses.

The program consists of two teaching manuals:

1. “Being Healthy”, which covers health skills such as using a thermometer, understanding prescriptions, communicating with health professionals, and Shared Decision-Making; and
2. “Staying Healthy”, which focuses on diet and exercise (healthy lifestyle).

The 16 Being Healthy and 14 Staying Healthy topics are listed in Table 1. Teachers will be given a suggested delivery plan, with advice that they may diverge from this, according to the interests and capabilities of their students. The main requirement for delivery is that classes cover all 10 “core” topics considered central to the health literacy learning objectives and linked to the quantitative assessments. For the remainder of lessons students and teachers can select topics of most interest and relevance to the class.

**Table 1:** Course content

Being healthy Teacher manual 1	Staying healthy Teacher manual 2
1.1 Taking temperature^a^	2.1 Getting involved
1.2 Checking medicine labels^a^	2.2 Food groups
1.3 Prescriptions	2.3 Food labels^a^
1.4 Dosage and timing	2.4 Nutritional information^a^
1.5 Health workers	2.5 Food temperature safety
1.6 Telling your doctor what is wrong^a^	2.6 Food date safety
1.7 Talking to your doctor^a^	2.7 What is a serve?^a^
1.8 Answering your doctor’s questions^a^	2.8 Budgeting
1.9 Immunisation and health screening	2.9 Understanding a diet
1.10 Asking questions^a^	2.10 Drinking enough fluids
1.11 Shared decision-making^a^	2.11 Heart Rate and Pulse
1.12 Completing medical forms	2.12 Being Active
1.13 Emergency services	2.13 Watch First Aid demonstrations
1.14 Advice from pharmacist	2.14 Follow written instructions
1.15 Saving lives	2.15 Talking on the telephone
1.16 Follow emergency instructions	Revision/Goal setting

^a^Core topic

Development and piloting of the teacher manuals is described elsewhere [41]. Following the pilot, the program was modified in response to participant feedback; and sent to Australian health experts (including partner organisations NSW Health, NPS Medicine Wise, NSW Clinical Excellence Commission) for review. The final manuals can be obtained from the corresponding author.

### LLN (standard LLN) program

Standard LLN students will complete the “standard” TAFE content for the units of study. This includes learning computer skills, employment skills, and other non-health related activities and is designed to develop core skills and confidence in language, and improve functional English language, literacy and numeracy skills.

### Sample size

The primary outcome for sample size calculation is the functional health literacy score used in our pilot study ([41]). We estimate that 86 students are required per group to detect a difference in the mean total score between the intervention and standard LLN groups of 0.5 standard deviation (SD) with 90 % power at the two-sided 5 % significance level. An established systematic review indicates that a half-standard deviation is the ‘threshold of discrimination’, or the minimally important difference, for changes to psychosocial outcomes in chronic disease assessment [42]. To allow for clustering within a class and using an intra-cluster correlation coefficient of 0.05 and an average class size of 10, the required sample size is 125 students. Allowing for 15 % loss to follow-up, we aim to recruit 150 participants in each arm (*n* = 300 in total; 15 pairs of classes of approximately 10 students per class). This sample size will also be sufficient to detect a difference in retention of at least 20 % between the intervention and standard LLN group, with 80 % power. Due to the unpredictability of TAFE enrolments, unequal cluster sizes are assumed.

### Procedures

The program will be delivered by adult literacy teachers -with support from community health workers in the health literacy arm to assist with health-related content. All (health literacy and standard LLN) teachers will be trained in study procedures by University of Sydney researchers, and supplied with a study manual outlining rationale, procedures and timelines. Baseline measures will be taken on entry into the program and the short-term follow-up assessment will be conducted on completion of the program. Assessments will be split over several teaching sessions to spread student workload and delivered face-to-face by the adult literacy teacher (see Fig. 1). Participants will be invited to a further data-collection session 6 months following course completion, where they will receive $20 for their time. Students who are unable to attend the session will be able to complete the questionnaire by phone or mail.

### Blinding

Students will be invited to participate in a research trial but will be blinded as far as possible to its aims. Students will be informed simply that the study is about a new adult literacy program, the “Living Literacy Program”, but will not be informed that its purpose is to evaluate a health literacy program. Detail about the purpose of the trial will be given in a debrief statement following the final data collection session (6 months after course conclusion).

### Measures

Outcomes will be evaluated among students and teachers using a mixed methods approach. Table 2 summarises all outcome measures and timing of data collection for the study.

**Table 2:** Data assessment schedule

	Baseline	Post intervention			
		Immed	2 months	6 months	6–12 months
Quantitative evaluation					
Demographics	X			X	
Health literacy measures • Single Item Literacy Screener (SILS) • Reading ability • Newest Vital Sign (NVS)	X				
Health skills	X	X			
Confidence (in health activities)	X	X		X	
Health Literacy Questionnaire	X	X		X	
Patient Activation Measure		X			
Shared Decision Making		X		X	
Student evaluation		X			
Healthy lifestyle (self-report) • Fruit and vegetable intake • Physical activity				X	
Health knowledge				X	
Qualitative evaluation					
Teacher interview - intervention			X		
Teacher interview - standard LLN			X		
Student interview - intervention			X		
Student interview - standard LLN			X		
Comm. health worker interview			X		
Education outcomes					
LLN outcomes		X			
Attendance at classes		X			
Enrolment in future TAFE classes					X

### I. Evaluation among students

#### Quantitative evaluation

##### Baseline measures

Include demographic and health literacy screening questions. Demographic information includes age, sex, country of birth, language spoken at home, and self-reported health status.

Health literacy includes: the Single Item Literacy Screener (SILS) “How often do you need to have someone help you when you read instructions, pamphlets, or other written material from your doctor or pharmacy?” [43]; self-report of reading ability “How would you rate your ability to read” [44], using a cut off of 3 “sometimes”/”okay” to denote poor literacy; Newest Vital Sign (NVS) [45] with minor modification to the food units presented in the stimulus to make it suitable for use in Australia.

We will take baseline measures of the main primary and secondary outcomes: functional health literacy skills (described below and in Additional file 1), confidence, and components of the Health Literacy Questionnaire (HLQ), described below.

##### Immediate post intervention outcomes

The primary outcome of functional health literacy skill will be assessed using a purpose designed measure informed by the intervention course core content (see Additional file 1):

- How to use a thermometer (score range 0–3).
- Interpretation of a medicine labels, (score range 0–5).
- Interpretation of a food label, 9 items (score range 0–10; not assessed at baseline).

Knowledge scores will be calculated using a marking scheme developed a priori (see Additional file 2). Health skills tasks will be assessed separately, as well as a combined outcome, which will be a weighted sum of the components.

Secondary outcomes will be:

1. Confidence in health skills −10 confidence items modified from [46], measured on a 5-point scale ranging from “extremely” to “not at all” confident.
  1. Telling the doctor what is wrong; 2. Understanding your doctor; 3. Reading and understanding medicine labels; 4. Filling out medical forms by yourself; 5. Preventing problems with your health; 6. Taking care of your family and friends’ health; 7. Reading and understanding food labels; 8. Planning healthy meals; 9. Using a thermometer; 10. Asking the doctor questions.
2. The Health Literacy Questionnaire (HLQ) [47] comprises 9 independent subscales to assess different aspects of health literacy. We selected 5 scales relevant to the learning objectives of the study intervention program (Having sufficient information to manage my health; Actively managing my health; Ability to engage with healthcare providers; Navigating the healthcare system; Understanding health information well enough to know what to do) rated on a 4-point scale or 5-point scale (25 items total).
3. The 13-item generic Patient Activation Measure [48] includes self-reported ability of the individual to enact skills of health literacy using items assessing patients’ skills and confidence in managing their health.
4. Student satisfaction with the health literacy program. As in [41] we will ask students to rate their course experience using 5-point Likert scale items: 1. overall rating (Excellent-Very poor); 2. if the course was (a) easy to understand, (b) helped them to understand their health (strongly agree-strongly disagree); 3. if they would recommend the course to family and friends (yes, definitely-definitely not).
5. Shared Decision Making. A purpose-developed 14-item curriculum-based Shared Decision Making knowledge questionnaire (see Additional file 3) will cover Shared Decision Making competencies including comprehension of Shared Decision Making terms and concepts, probabilities and risk information. To assess the question asking component of the Shared Decision Making module we will ask all students to list (free-response) questions they considered important to discuss with their doctor, and code responses using content analysis. We will ask students who received the intervention to recall the AskShareKnow questions (free response), and analyse data following the method used by Shepherd et al. [37].

Six-month follow-up measures:

As assessed at baseline and immediately post intervention

1. Confidence.
2. Health Literacy Questionnaire.
3. Shared Decision Making.
  - decisional conflict using the SURE scale [49].
  - decision making preferences using the Control Preferences scale [50].
  - recall of the three AskShareKnow questions, use in health-care consultations based on self-report by participants, and comments on AskShareKnow materials (intervention only).

Additional measures

4. Health knowledge using a 12-item curriculum based measure to assess retention of core components of health knowledge. 7 items related to health information and services, and 5 items were about serve sizes. See Additional file 4.
5. Healthy lifestyle using self-report questions of daily: fruit and vegetable intake (number of serves); walking, and moderate and hard physical activity. These measures were taken from the 45 and Up Study [51].

6–12 month follow-up

1. Program retention rate: data on the proportion of adults enrolled in the program who complete the entire program, and proportion of classes attended will be collected.
2. Enrollment in further TAFE adult education programs 12 months following course completion.

These final two endpoints will be assessed by enrolment records collected routinely by TAFE.

#### Qualitative evaluation

Semi-structured interviews will be carried out with a purposively selected sub-sample of students (*n* = 30) who participated in the program to examine their experiences of the program and its impact on their knowledge, understanding, confidence and capabilities. The sample will include students selected from regional and metropolitan areas and varied levels of participation (from non-completion to full completion). Interviews will be carried out face to face and by telephone, audio recorded and transcribed verbatim. Data will be analysed thematically using Framework Analysis.

### II. Evaluation among teachers and community health workers

#### Quantitative evaluation

Intervention teachers will be asked to comment on and rate the teaching resources using a 5 point Likert scale (very good – very poor) with space for additional comments. This measure is included within the teaching manuals and is intended to be completed following each lesson.

#### Qualitative evaluation

Telephone interviews will be conducted with all intervention teachers and community health workers, and a subset of standard LLN teachers, on completion of the program. The interviews will explore their experiences and views on the content, delivery and success of the program, challenges faced, suggestions for improvement and how adult literacy teachers and health professionals worked together to deliver the program. Standard LLN teachers will be asked about experiences with the class and possibilities for contamination. As before interviews will be audio recorded, transcribed verbatim and thematically analysed.

#### Statistical analysis

The primary analysis will be by intention to treat. We will compare outcomes between the intervention and standard LLN arms for the primary outcome using a regression analysis that will account for clustering using appropriate methods [52]. We will use linear, logistic or ordinal logistic regression analyses as appropriate.

We will also conduct secondary analyses that adjust for baseline scores on the measures (where available), and health literacy and numeracy (measured with NVS). We will conduct these analyses separately. We will also conduct the same set of analyses for the primary outcomes, but using the intervention as actually received.

The statistician will be blinded to the allocated group definitions (i.e., health literacy vs standard LLN) until completion of the statistical analysis.

#### Quality assurance

Data will be collected on paper-based questionnaires and entered into a central computerised database. Ten percent 10 % of records will be manually checked against the paper copies. For knowledge items that require coding and scoring, double-marking will be conducted to ensure consistency.

#### Project reference committee

A cross-sectoral reference group will oversee the research, comprising representatives from partner organisations (National Prescribing Service, Clinical Excellence Commission, NSW Health, TAFE NSW), the Chief/Partner Investigators, and NSW Health Literacy Network, a national group chaired by the NSW CEC involving state and national stakeholders from health, adult education, and consumer representatives; and external experts in health and adult education. The target group (i.e. adult learners with low literacy) will be represented by an appointed ‘consumer representative’ and by being actively involved in the development of the health literacy program to ensure the content and structure of the program is tailored to their needs, preferences and skill level.

#### Expected outcomes of the study

This research aims to develop and evaluate a novel adult health literacy training program to improve both language, literacy, and numeracy skills, together with specific health literacy competencies among socially disadvantaged Australian adults. It will assess the achievement of key literacy and health literacy outcomes when delivered under “real life” conditions at selected Australian adult education centres, and provide evidence on how the program can be implemented in varied circumstances. There is no existing model for collaboration in Australia between statutory adult education and public health organisations that systematically addresses both health and education outcomes. This project will enable us to test this cross-sector collaboration and provide high quality data to inform both education and health policy decisions.

## Discussion

This study seeks to demonstrate effectiveness of a health literacy program embedded in an adult basic education setting, in improving both health literacy and adult education outcomes. In the process of doing so, it seeks to build partnerships between health and education agencies to find new ways to deliver targeted health messaging to these difficult-to-access populations. The evaluation trial will provide insight into translating and scaling up health literacy education for vulnerable populations with low literacy, including linguistically and culturally diverse adults, and durability of any observed gains. We hope that the results of this trial will influence policy makers to open pathways for health and education agencies to work together to serve the needs of these disadvantaged populations – which may reduce some of the costs to the community of limited health literacy.

### Registration details

The trial is registered with the Australian New Zealand Clinical Trials Registry (registration no. ACTRN 12616000213448).

## Additional files

Additional file 1: Stimuli for functional health skills measures. (PDF 430 kb) Additional file 2: Scoring scheme for health skills measures. (PDF 101 kb) Additional file 3: Shared decision-making (immediate follow-up). (PDF 352 kb) Additional file 4: 6 month knowledge measures (12 items). (PDF 230 kb)

## Abbreviations

ACSF: Australian Core Skills Framework
CALD: culturally and linguistically diverse
CEC: Clinical Excellence Commission
FCE: Functional Context Education
HLQ: Health Literacy Questionnaire
LLN: language, literacy, and numeracy
NESB: non-english speaking background
NPS: National Prescribing Service
NSW: New South Wales
NVS: Newest Vital Sign
REALM: Rapid Estimate of Literacy in Medicine
SILS: Single-Item Literacy Screener
TAFE: Technical and Further Education
TOFHLA: Test of Functional Health Literacy in Adults
UK: United Kingdom
US: United States
