# Supplementary material for: Evaluation of an Australian health literacy training program for socially disadvantaged adults attending basic education classes: study protocol for a cluster randomised controlled trial
Source: BMC Public Health. 2016 May 27;16:454. doi: 10.1186/s12889-016-3034-9 (PMC4884424; doi:10.1186/s12889-016-3034-9)
Supplement: Additional file 1: — Stimuli for functional health skills measures. (PDF 430 kb) [file 12889_2016_3034_MOESM1_ESM.pdf]

Additional file 1 Stimuli for functional health skills measures

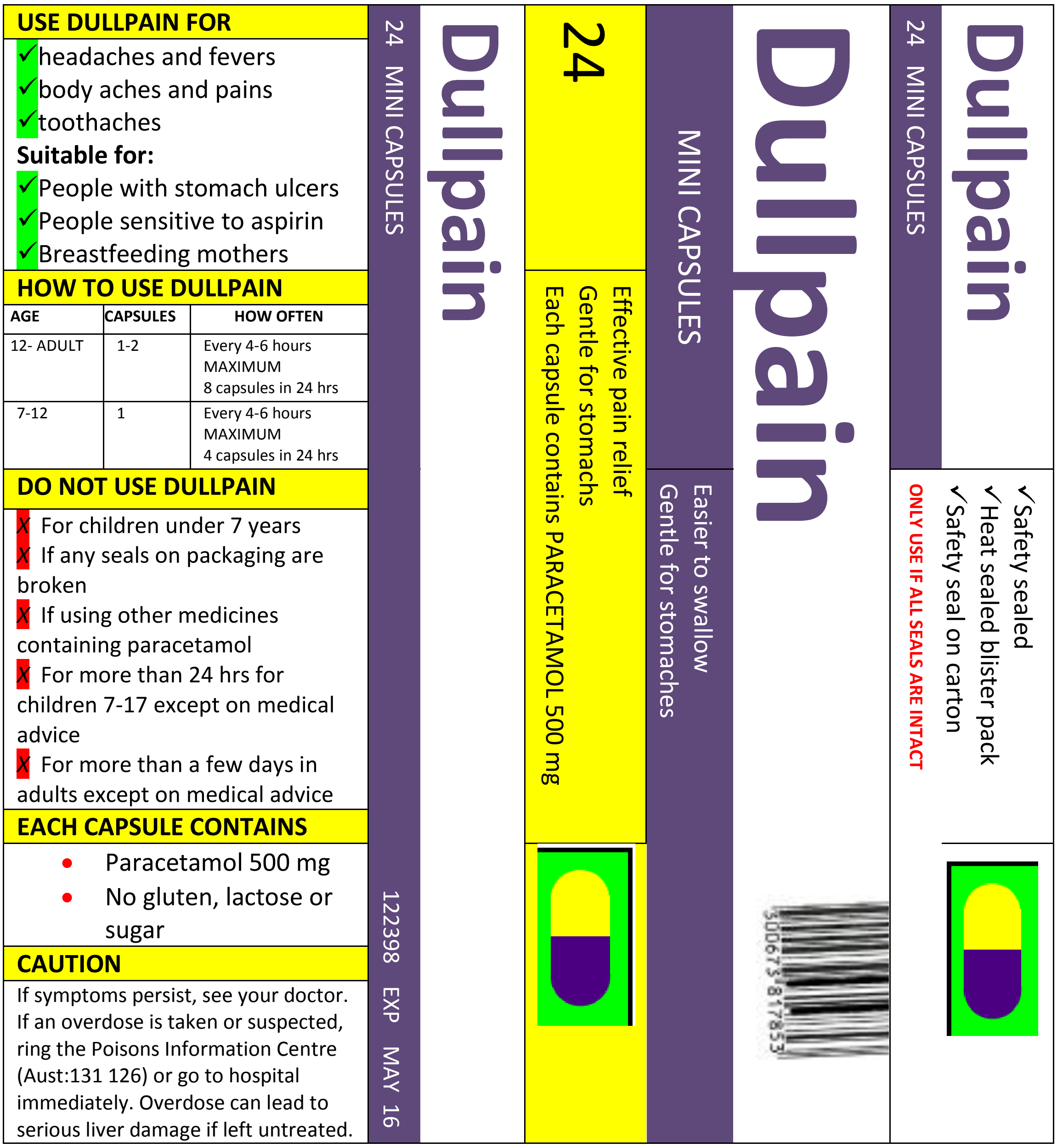

1. What temperature is shown on thermometer 1?<br><br>__________	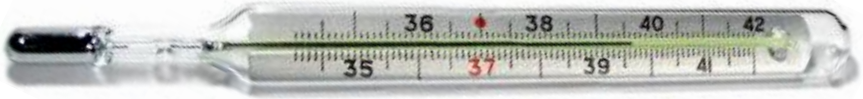
2. This morning I took my temperature with thermometer 2.<br><br>This is the reading I got. Is this ok?<br><br>❑ No ❑ Yes	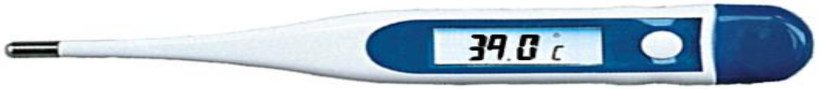
3. On thermometer 3 write what temperature you think is normal for a healthy adult.	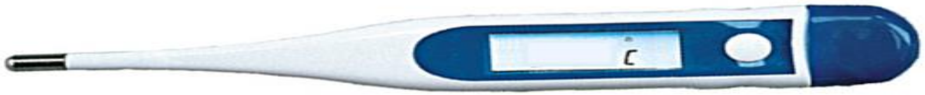

Foody Feast

Soup in a Mug

Purse size sachets

Great for work. Great for play.
Use any time of day.

50g
2 Sachets

Potato and leek

Ingredients

Vegetables, creamer [vegetable oil, glucose syrup, milk protein, mineral salts (339, 450)], chives, maltodextrin (from wheat), salt, flavour enhances (621, 627), wheat fibre, mineral salt (potassium chloride), vegetable oil, garlic, hydrolysed corn protein, flavours, emulsifier (471), colour, spice extract, preservative (222), food acid (citric)

**NUTRITION INFORMATION**		
Servings per package: 2<br>Serving size: 300mL		
	Avg Qty per Servin g	Avg Qty per 100mL
Energy	493kJ	164kJ
Protein	2.5g	0.8g
**Fat, Total**	**2.4**	**0.8g**
-saturated	2.2g	0.7g
Carbohydrate	15.6g	5.2g
-sugar	1.2g	0.4g
Dietary fibre	1.7g	0.6g
Sodium	595m g	198mg
Potassium	450m g	150mg

Directions

1. **Empty contents of one sachet into a 300 mL mug.**

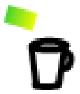

2. **Fill with boiling water and stir for 15-20 seconds.**

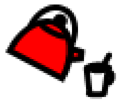

Best Before 12/04/13

Product Description: Ice cream

NUTRITION INFORMATION

Servings per package: 4

Serving size: 100mL

	Average quantity per serving
Energy	1050kJ
Calories	250 kcal
Protein	4g
Fat, total	13g
- saturated	9g
- monounsaturated	0g
- polyunsaturated	3g
- trans	1g
Carbohydrate	30g
- sugars	23g
Fibre	0g
Sodium	55mg

Ingredients: Cream, Skim Milk, Sugar, Egg, Stabilisers (Guar Gum), Peanut Oil, Vanilla Extract (0.05%)
