# Supplementary material for: Evaluation of an Australian health literacy training program for socially disadvantaged adults attending basic education classes: study protocol for a cluster randomised controlled trial
Source: BMC Public Health. 2016 May 27;16:454. doi: 10.1186/s12889-016-3034-9 (PMC4884424; doi:10.1186/s12889-016-3034-9)
Supplement: Additional file 2: — Scoring scheme for health skills measures. (PDF 101 kb) [file 12889_2016_3034_MOESM2_ESM.pdf]

Additional file 2 Scoring scheme for health skills measures

Questions	Marking scheme	Max score
**Medicine labels (baseline and immediate follow-up)**		
1. When does the medicine expire?	May 2016 = 1 (response 3)<br>Zero for any other responses	1
2. I have a stomach ulcer. Can I use this medicine?	Yes = 1 (response 2)<br>Zero for any other responses	1
3. My son is 6 years old. Can he use this medicine?	No = 1 (response 1)<br>Zero for any other responses	1
4. I took 2 capsules at 10am. When can I take another 2?	2pm – 4pm =1<br>14:00 – 16:00 /2pm / 3pm / 4pm = 1<br>Zero for 2am-4am<br>Zero for 4-6 hours<br>Zero for 2pm-6pm<br>Zero for any other responses	1
5. My 3 year old son found a packet and has eaten 6 capsules. What should I do?	Call the Poison Information Centre or go to hospital immediately = 1<br>Mention “hospital” “poisons information” = 1<br>Zero for “ambulance” “go to doctor”	1
	**Total**	**5**
**Taking temperatures (baseline and immediate follow-up)**		
1. What temperature is shown on thermometer 1?	39 – 40 = 1<br>Zero for any other values	1
2. This morning I took my temperature with thermometer 2. This is the reading I got. It this ok?	No = 1<br>Zero for any other responses	1
3. On thermometer 3 write what temperature you think is normal for a healthy adult.	35.8 – 37.4 = 1<br>96.4 - 99.3F = 1<br>Zero for any other values	1
	**Total**	**3**

Food labels and Nutritional information (immediate follow-up only)		
1. Do you look at food labels when you buy packaged food?		NA
2. What kind of food is this?	soup, potato and leek soup, vegetable soup = 1<br>Zero for any other responses e.g. dried, sachet	1
3. What is the main ingredient?	Vegetables = 1<br>Zero for potato & leek	1
4. How do you make this food?	Similar to correct answers.<br>Empty one sachet into a mug= 1<br>Fill with boiling water and stir = 1	2
5. If this package was being sold next July would you buy it? Why/Why not?	Similar to correct answers (1)<br>Reason why (expiry date)= 1<br>No + reason incl expiry date =1<br>Reason (exp date) implying 'no' (not explicitly stating 'No') = 1<br>No (without stating reason) = 0<br>No + reason not expiry date = 0<br>"no good" = 0<br>Yes = 0<br>No= 0	1
6. What are kilojoules?	A measurement of energy. Or explain that food high in kilojoules are high energy foods<br>Anything with energy = 1<br>1000 units of joules = 0<br>Fat/weight/calorie = 0	1
7. How many kilojoules are in a serve of this food?	493 kj = 1<br>Zero for any other value	1
8. How do you find out if this food is high in salt?	Look at sodium or nutrition information table or cite actual figure = 1<br>Check ingredients/label = 0<br>Zero for any other response	1
9. Why would this be important to check?	Salt/sodium can cause high blood pressure; or mention heart, kidneys, diabetes, blood pressure = 1<br>Healthy / no salt diet = 0<br>Zero for any other response	1
10. What else would you check to make sure this food was healthy?	Any of the following: fat, sugar, any allergy foods (nuts, dairy, wheat, gluten), added flavours/ colours/preservatives/ numbers in the ingredients = 1<br>expiry date / ingredients / nutrition information = 0<br>Salt/sodium = 0	1
	**Total**	**10**

Newest Vital Sign (baseline only)

	Published criteria	Accepted responses	Max
1. How many calories (kcal) will you eat if you eat the whole container?	1,000 KCAL or 1,000 CALORIES<br>4200kJ	1000 calories<br>1000 kcal<br>4200 kj<br>1000 g/c/ccal/mkl/ml/	1
2. If you are advised to eat no more than 60 grams of carbohydrate for dessert, what is the maximum amount of ice cream you could have?	Two servings (or anything up to 2 servings) OR Half the container (or any amount up to half the container) OR 200 ml (or any amount up to 200 ml).	2 ice creams/2 scoops/1 scoop/200ml/200g/ ½ container<br>Zero for 2g/13g/180g / 30g / 50g / 60/2 cups / 2	1
3. Imagine that your doctor advises you to reduce the amount of saturated fat in your diet. You usually have 42g of saturated fat each day, some of which comes from one serving of ice cream. If you stop eating ice cream, how many grams of saturated fat would you be eating each day?	33g	33g<br>Zero for any other	1
4. If you usually eat 2500 calories each day, what percentage of your daily calories (kcal) intake will you get if you eat one serving of ice cream?	10% or 1/10th	0.1<br>10%	1
5. Is it safe for you to eat this ice cream?	No	No	1
6. Why or why not?	Because it contains peanut oil/peanuts/nuts	Peanuts/peanut oil<br>Allergic to peanuts<br>Zero for “allergic”	1
		**Total**	6
