# Supplementary material for: Evaluation of an Australian health literacy training program for socially disadvantaged adults attending basic education classes: study protocol for a cluster randomised controlled trial
Source: BMC Public Health. 2016 May 27;16:454. doi: 10.1186/s12889-016-3034-9 (PMC4884424; doi:10.1186/s12889-016-3034-9)
Supplement: Additional file 3: — Shared decision-making (immediate follow-up). (PDF 352 kb) [file 12889_2016_3034_MOESM3_ESM.pdf]

Additional file 3 Shared decision-making (immediate follow-up)

Please put a cross in the box that you think has the right answer.

Question	Multiple choice responses (correct is bold)
1. What is shared decision making?	Doctor decides<br>**Doctor and patient decide together**<br>Patient decides<br>Doctor and nurse decide together
2. Which word is most like the word 'options'?	Advantages (something good)<br>Lists<br>**Choices**<br>Total<br>Treatment<br>Disadvantages or Problems
3. Which word is most like the word 'benefit'?	**Advantages (something good)**<br>Lists<br>Choices<br>Total<br>Treatment<br>Disadvantages or Problems
4. Which word is most like the word 'harm'?	Advantages (something good)<br>Lists<br>Choices<br>Total<br>Treatment<br>**Disadvantages or Problems**
5. Which of the following numbers represents the biggest risk of getting a disease?	1 in 100<br>1 in 1000<br>**1 in 10**
6. Which of the following numbers represents the biggest risk of getting a disease?	1%<br>**10%**<br>5%
7. If the chance of getting a disease is 20 out of 100, this would be the same as a _______% chance of getting the disease.	free response (20)

Here is some information about a new vaccine – Vaccine X. Please read the information carefully and look at the picture.

There is a new vaccine available, called Vaccine X. It helps to prevent Disease Y. There were some mild side effects of the vaccine, including: (1) Headaches, (2) Diarrhea, and (3) Fever. The picture below shows how likely each of these side effects are.

Side-effects of Vaccine X

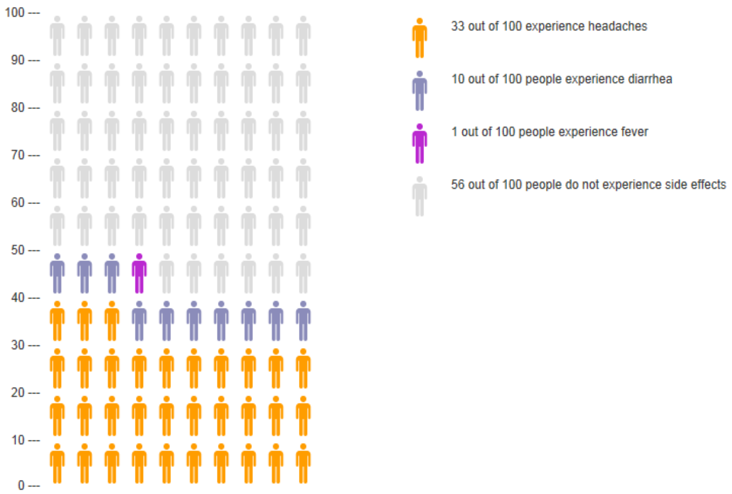

8. Which side-effect was most likely?	**Headaches**<br>Diarrhea<br>Fever	
9. Which side effect was least likely	Headaches<br>Diarrhea<br>**Fever**	
10. People were more likely to:	Experience side-effects<br>**Not experience side-effects**	
11. Out of 100 people, how many people will have a fever?	33<br>10<br>**1**<br>56	
12. Out of 100 people, how many people will have headaches?	**33**<br>10<br>1<br>56	
13. Choose one word to describe the risk of fever	Certain<br>Very likely<br>Likely<br>**Possible**	**Unlikely**<br>**Very unlikely**<br>Impossible
14. Choose one word to describe the risk of headaches	Certain<br>Very likely<br>**Likely**<br>**Possible**	Unlikely<br>Very unlikely<br>Impossible
