# Supplementary material for: Evaluation of an Australian health literacy training program for socially disadvantaged adults attending basic education classes: study protocol for a cluster randomised controlled trial
Source: BMC Public Health. 2016 May 27;16:454. doi: 10.1186/s12889-016-3034-9 (PMC4884424; doi:10.1186/s12889-016-3034-9)
Supplement: Additional file 4: — 6 month knowledge measures (12 items). (PDF 230 kb) [file 12889_2016_3034_MOESM4_ESM.pdf]

Additional file 4 6 month knowledge measures (12 items)

Health Quiz. Please choose the answer that you think is the most correct.

1. My pharmacist asks if I want the generic brand of my prescribed medicine. What does generic brand mean?	A different brand of your medicine that has fewer doses<br>A different brand of your medicine that is stronger<br>A different brand of your medicine that is weaker<br>**A different brand of your medicine that has the same active ingredient and can cost less**	
2. What does PBS refer to?	A health care professional you would go to if you wanted advice on your diet<br>A disease that causes high levels of glucose in the blood<br>**The Australian government program that helps to pay some of the cost of your medicine**<br>The organisation in Australia that offers free bowel cancer screening	
3. What information will you not find on a medicine label?	Side-effects<br>Ingredients<br>**Contact information of your closest GP**<br>What age group can take the medicine	
4. In Australia, who can be helped by the PBS?	People over 55 years old only<br>People with private health insurance only<br>Australian residents with chronic diseases (such as cancer) only<br>**Australian residents who have a Medicare card**	
5. The chances of getting side effects from Medicine X is 76%. Choose one word that describes the risk of getting side effects.	Certain<br>**Very likely**<br>**Likely**<br>Possible	Unlikely<br>Very unlikely<br>Impossible
6. Your doctor asks you to 'wait and watch'. What does 'wait and watch' mean?	When you wait in the waiting room and watch TV<br>**When you carefully watch your symptoms without having any treatment**<br>When you carefully watch your symptoms whilst you are having treatment<br>When you wait for your test results to come back	
7. My father has diabetes and needs to be careful about what he eats. Who can help him plan his meals?	Paediatrician<br>**Dietician**<br>Optometrist<br>Psychologist	

Serves quiz

Please mark true or false

8.	1 whole watermelon = 1 serve of fruit	False
9.	2 large eggs = 1 serve of protein	True
10.	2 slices of wholemeal bread = 1 serve of grain foods	False
11.	1 cup of tea with milk (but no sugar) = 1 serve of dairy	False
12.	½ glass of red wine = 1 serve of fruit	False
